# Minimally Invasive Treatment of Uterine Myoma in a Patient With Thrombocytopenia: A Case of Uterine Artery Embolization Followed by Laparoscopic Surgery

**DOI:** 10.7759/cureus.104333

**Published:** 2026-02-26

**Authors:** Yumiko Kitago, Hyo Kyozuka, Toma Fukuda, Riho Yazawa, Yasuhisa Nomura

**Affiliations:** 1 Obstetrics and Gynecology, Ohta Nishinouchi Hospital, Koriyama, JPN

**Keywords:** minimally invasive therapy, thrombocytopenia, total laparoscopic hysterectomy, uterine artery embolization, uterine fibroid

## Abstract

Uterine fibroids are noncancerous tumors that affect women of reproductive age. Traditional surgical interventions for uterine fibroids require larger incisions and may require longer hospital stays and recovery times than minimally invasive therapies, such as laparoscopic myomectomy, hysteroscopic myomectomy, and uterine artery embolization (UAE). Preoperative UAE blocks the blood supply to fibroids and reduces intraoperative blood loss. This report describes the successful treatment of a patient with uterine fibroids and thrombocytopenia by UAE followed by laparoscopic surgery. The patient presented with sudden massive genital bleeding and visited our hospital immediately. UAE was performed because of the potential risk of surgical management. Three months after the UAE, a total laparoscopic hysterectomy (TLH) was performed to treat uterine fibroids. TLH was completed without bleeding. As a minimally invasive approach, UAE combined with TLH benefits women at a high risk of intraoperative bleeding, allowing the successful completion of a low-risk procedure without complications.

## Introduction

Uterine fibroids (leiomyomas) are benign tumors arising from the proliferation of uterine smooth muscle cells and commonly affect women of reproductive age [1]. Common symptoms include pelvic pressure, dysmenorrhea, heavy menstrual bleeding, urinary frequency, and infertility [2]. Conventional surgical management, such as open abdominal hysterectomy or laparotomic myomectomy, may require longer hospitalization and recovery compared with minimally invasive options, including laparoscopic or hysteroscopic myomectomy and uterine artery embolization (UAE) [3-5]. In selected patients, UAE can be used to achieve hemostasis and/or reduce bleeding risk before subsequent surgical management. Here, we report a patient with symptomatic uterine fibroid complicated by thrombocytopenia who was successfully managed with staged UAE followed by laparoscopic surgery.

## Case presentation

**Figure 1:**
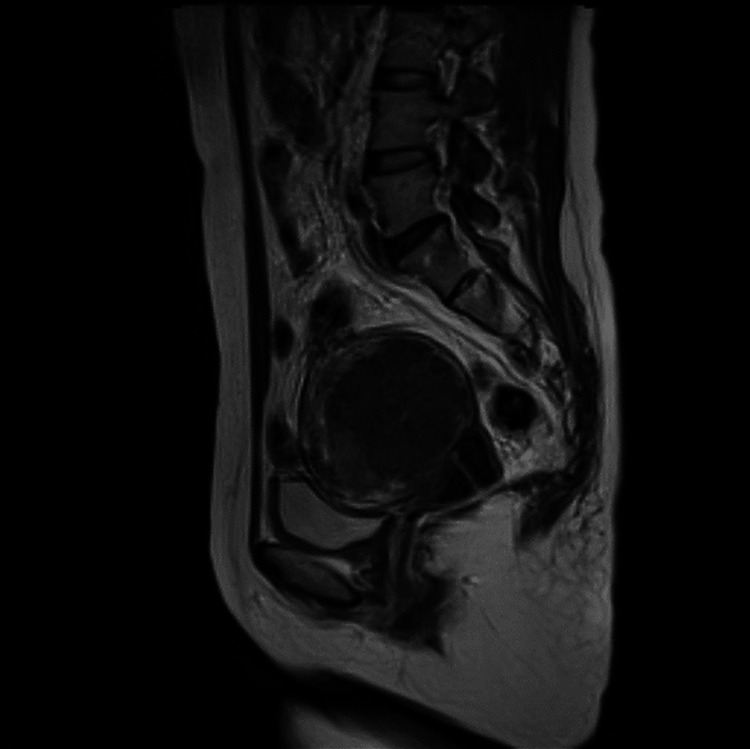
MRI finding at first presentation A well-defined ~7 cm leiomyoma was identified in the uterine fundus on the T2-weighted image.

A 40-year-old Japanese woman with a history of three spontaneous vaginal deliveries presented with severe menstrual bleeding. Her medical history included splenomegaly and chronic thrombocytopenia diagnosed two years earlier, and she was not taking any medications. Pelvic magnetic resonance imaging (MRI) revealed a well-defined intramural leiomyoma measuring ~7 cm in the uterine fundus (Figure 1). Initial laboratory testing showed anemia (hemoglobin, 10.4 g/dL) and thrombocytopenia (platelet count 2.5 ×10^4/μL). Oral dienogest at a dose of 2 mg/day was initiated for bleeding control.

**Figure 2:**
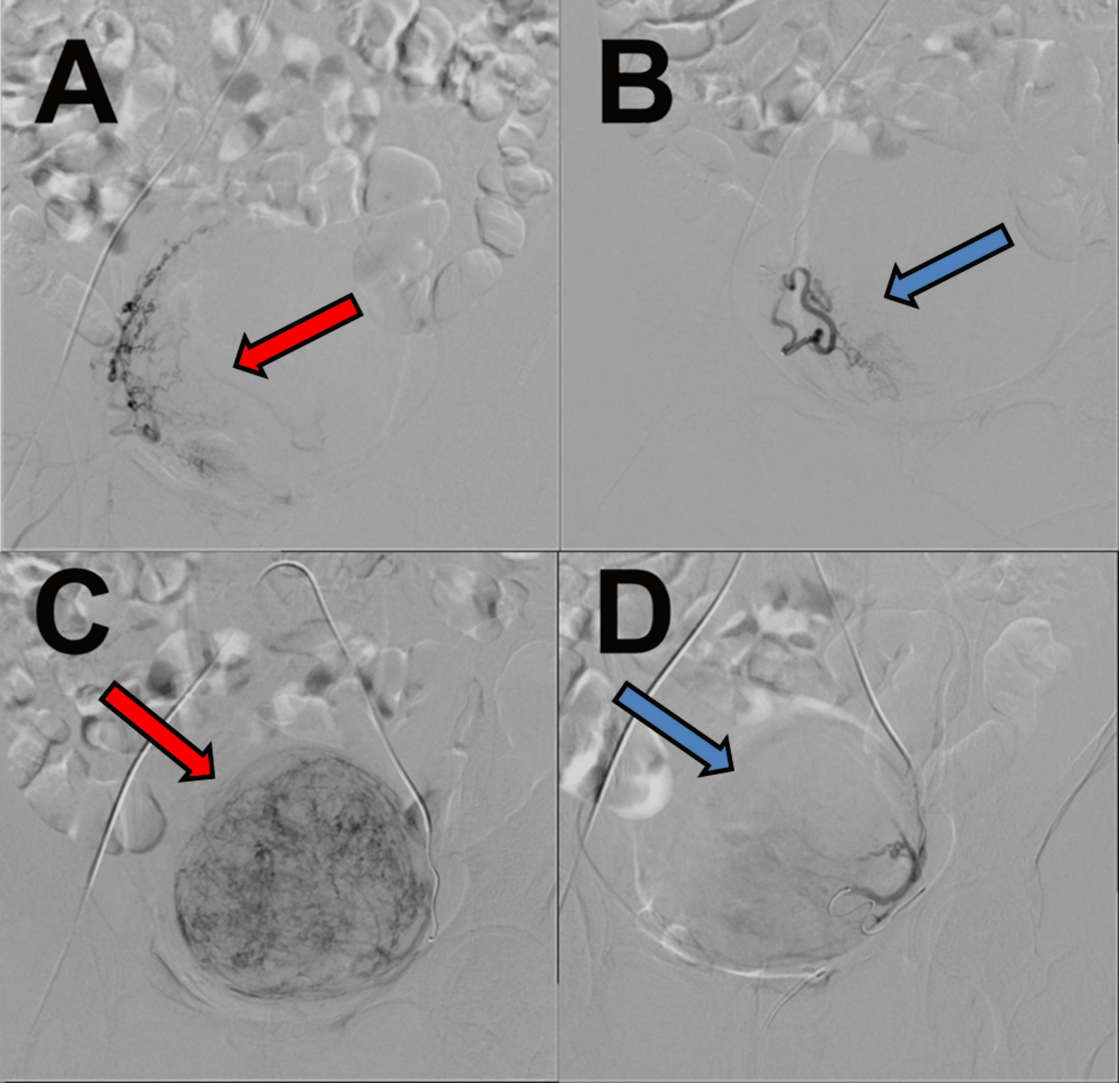
Angiographic findings before and after uterine artery embolization A: Right uterine artery angiography before embolization. The red arrow indicates a vascular area supplying the uterine fibroid (tumor blush). B: Right uterine artery angiography after embolization with a 2-mm gelatin sponge. The blue arrow indicates a reduction of tumor vascularity. C: Left uterine artery angiography before embolization. The red arrow indicates a prominent tumor blush. Compared with panel A, the left uterine artery supplied more blood flow to the fibroid, and the entire fibroid contour was more clearly visualized, suggesting that the left uterine artery was the dominant feeder. D: Left uterine artery angiography after embolization with a 2-mm gelatin sponge. The blue arrow indicates marked reduction of tumor vascularity.

Shortly after starting dienogest, she developed sudden massive genital bleeding and was emergently admitted. The shock index was >1, and laboratory testing demonstrated persistent thrombocytopenia and hypofibrinogenemia (Table 1). Given ongoing hemorrhage with an unstable bleeding risk profile and a large fibroid, emergency UAE was performed for hemostasis, with a plan for interval total laparoscopic hysterectomy (TLH). Emergency UAE was performed under local anesthesia via the right common femoral artery. After placement of a 5-Fr arterial sheath, selective pelvic angiography was performed using a 5-Fr diagnostic catheter. A 2.2/2.9-Fr microcatheter was advanced for selective catheterization of each uterine artery. Nonionic iodinated contrast medium was used, with a total contrast volume of 100 mL. For embolization, a gelatin sponge (Serescue® Astellas Pharma) was used as the embolic agent. The 2-mm gelatin sponge material was finely cut with scissors before use. Bilateral UAE was then performed, and embolization was continued until a marked reduction of uterine artery flow was achieved (Figure 2).

**Table 1:** Laboratory findings over the clinical course NA: not available; UAE: uterine artery embolization; TLH: total laparoscopic hysterectomy; WBC: white blood cell count; PT-INR: prothrombin time-international normalized ratio; APTT: activated partial thromboplastin time

Parameter	Unit	Reference range	Initial laboratory test	Emergent admission (pre-UAE)	Immediately after UAE	Admission for TLH	After platelet transfusion (pre-op)	Immediately after TLH
Hemoglobin	g/dL	11.6-14.8	10.4	11.5	13.5	14.1	NA	11.8
Platelet count	×10^4/μL	15.8-34.8	2.5	2.3	5.3	5.0	6.5	5.3
WBC	/μL	3300-8600	4400	6500	7100	3800	NA	4400
PT-INR		0.8-1.2	NA	1.49	1.26	1.24	NA	NA
APTT	sec	24-37	NA	44.8	28.7	30.9	NA	NA
Fibrinogen	mg/dL	150-400	NA	120	163	NA	NA	NA
D-dimer	μg/mL	<1.0	<0.5	2.2	1.2	NA	NA	NA

**Figure 3:**
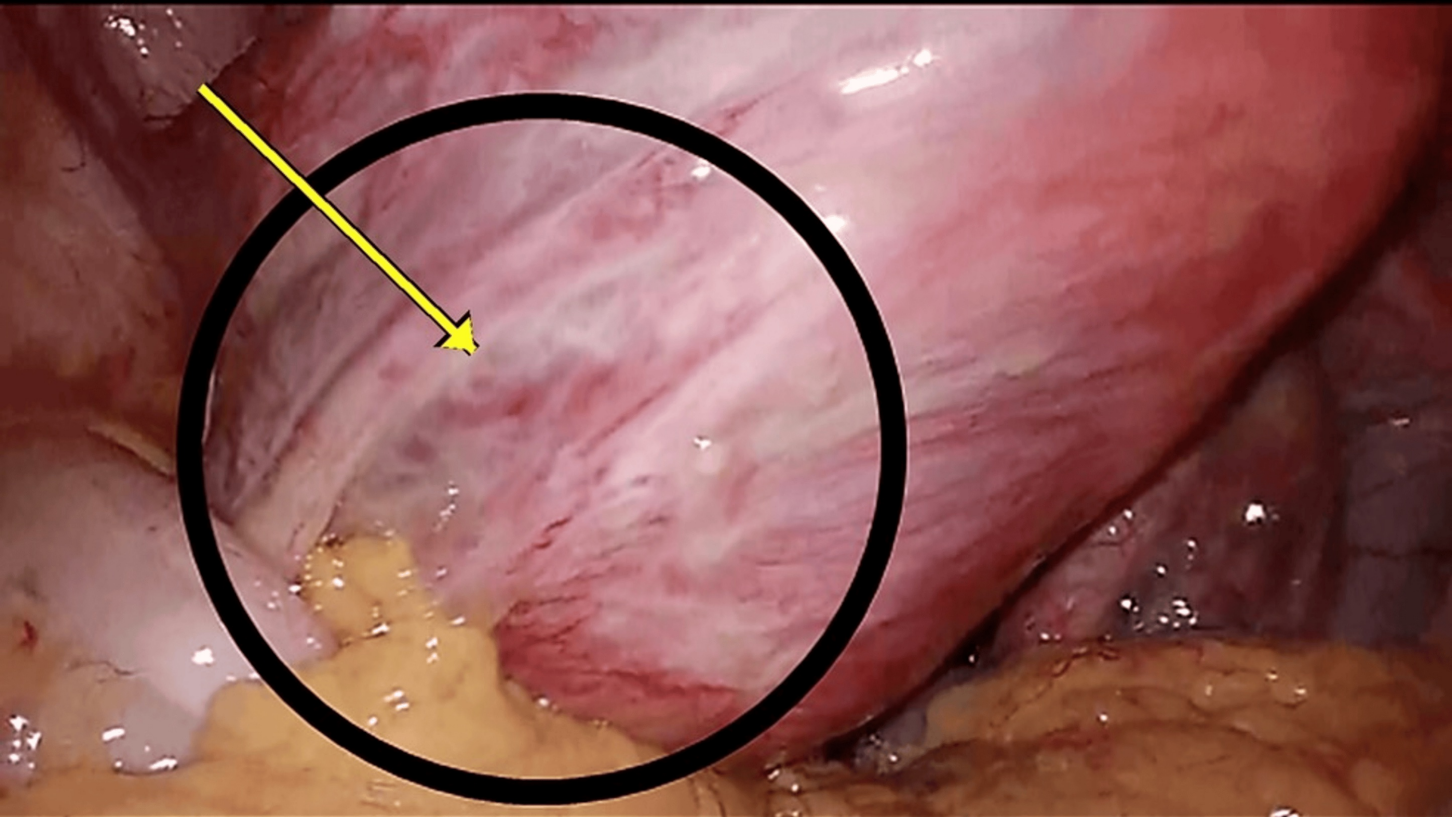
Laparoscopic findings White discoloration of the vessels of the left uterine posterior serosa, supplied by the left uterine artery, was observed, suggesting blood supply from the left uterine artery to the uterus was reduced (black circle).

**Figure 4:**
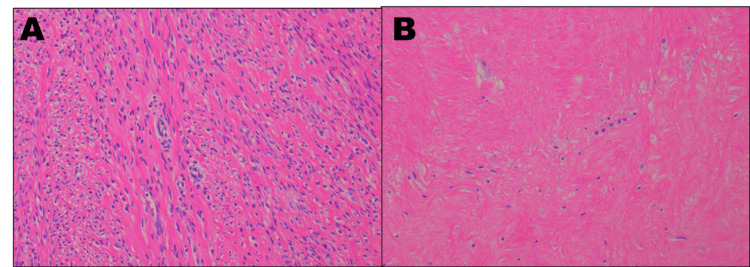
Pathological findings (hematoxylin and eosin staining) A: No remarkable atypia or mitotic figures were observed in the proliferating smooth muscle cells (×200). B: Most spindle cells are enucleated, likely due to the effect of UAE, leaving relatively few viable cells (×200). UAE: uterine artery embolization

Bleeding ceased after UAE, and she was discharged two days later. No further genital bleeding occurred during the three-month interval. She was readmitted for planned TLH three months after UAE. The preoperative platelet count remained approximately 5 ×10^4/μL (Table 1). Immediately before surgery, platelet transfusion increased the platelet count to 6.5 ×10^4/μL. Laparoscopy revealed an enlarged uterus with a fist-sized fibroid and blanching/whitening of the serosal vasculature (Figure 3). No interval MRI was obtained before TLH; therefore, angiographic reduction/disappearance of tumor blush after UAE (Figure 2) was used as the radiologic evidence of decreased fibroid vascularity. TLH was completed without excessive bleeding, and she was discharged three days postoperatively without complications. Histopathology confirmed leiomyoma without significant atypia or increased mitotic activity. Most spindle cells were enucleated, consistent with ischemic change after UAE, with relatively few viable cells remaining (Figure 4). At 1.5 years after TLH, she continued hematology follow-up with platelet counts around 50,000/μL, and splenectomy was being considered to improve thrombocytopenia.

## Discussion

Recent gynecologic management has increasingly focused on completing treatment using minimally invasive approaches whenever feasible. In the present case, the patient developed severe uterine bleeding due to a fibroid in the setting of chronic thrombocytopenia and presented in shock. Emergency UAE was performed to achieve hemostasis, followed by interval TLH as definitive treatment. This staged strategy allowed bleeding control in the acute phase and subsequent minimally invasive surgery after stabilization.

Large, multiple, or unfavorably located myomas may be associated with increased technical difficulty and bleeding risk during surgical treatment [6]. UAE was initially introduced in gynecologic/obstetric practice as a hemostatic procedure and later became established as a minimally invasive treatment option for symptomatic uterine fibroids [6,7]. Previous reports have suggested that preoperative UAE may improve the perioperative course in selected surgical cases, including those with large or anatomically challenging fibroids [8-10]. In the current case, thrombocytopenia and severe active bleeding were major considerations in treatment planning. We therefore selected emergency UAE for hemostasis and risk reduction, followed by TLH after clinical stabilization.

We did not obtain interval MRI before TLH; therefore, direct radiologic assessment of interval fibroid size change was not available. However, post-embolization angiography demonstrated marked reduction/disappearance of tumor blush (Figure 2), which was used as the available radiologic evidence of decreased fibroid vascularity. In addition, intraoperative blanching/whitening of the serosal vasculature (Figure 3) and the absence of excessive bleeding during TLH were clinically consistent with reduced uterine/fibroid perfusion after UAE.

Although thrombocytopenia is not specific to TLH, low platelet counts may increase perioperative bleeding risk and complicate hemostasis during laparoscopic hysterectomy, particularly in patients with active hemorrhage or coagulation abnormalities. In this case, perioperative management included staged hemostatic treatment (UAE) and preoperative platelet transfusion to increase the platelet count before TLH.

To the best of our knowledge, this is a rare reported case of severe uterine fibroid-related bleeding in a patient with chronic thrombocytopenia managed with emergency UAE followed by TLH. However, because this is a single case report, the effectiveness and generalizability of this strategy should be interpreted cautiously.

The optimal embolic agent for preoperative UAE in this setting remains uncertain. Prior reports have used different embolic materials, including resorbable gelatin particles and trisacryl gelatin microspheres, depending on treatment goals and local practice [9,10]. In the present case, a gelatin sponge was selected for emergency hemostasis as part of a staged treatment strategy. Gelatin sponge embolization can reduce arterial inflow and promote thrombosis, but recanalization may occur over time because it is a temporary embolic material [11-14]. In our case, angiography showed immediate reduction of tumor vascularity, and intraoperative findings three months later suggested persistent reduction in perfusion; however, the timing of surgery after gelatin sponge embolization should be individualized because the durability of occlusion may vary.

Particulate embolic agents such as polyvinyl alcohol (PVA) particles and trisacryl gelatin microspheres (Embosphere®, Merit Medical Systems, Utah, USA) provide more durable distal embolization and are widely used for UAE in symptomatic fibroids. However, stronger or more distal embolization may also increase the risk of ischemic complications, including non-target embolization, tissue necrosis/infection, pain, and potential effects on ovarian function [15]. Therefore, the choice of embolic material should be based on the clinical objective (emergency hemostasis vs. definitive fibroid devascularization), timing of planned surgery, vascular anatomy, and patient factors, rather than assuming superiority of a single agent. In the current case, sustained flow reduction was achieved with gelatin sponge embolization, and the subsequent TLH was completed without excessive bleeding.

## Conclusions

Preoperative UAE followed by TLH may offer a safe, minimally invasive strategy for women with uterine fibroids who are at high risk of surgical bleeding due to thrombocytopenia. In emergency settings, UAE can provide rapid hemostasis and a bridge to definitive surgery. Further accumulation of cases is needed to optimize the choice of embolic materials and the ideal interval between UAE and surgery.
